# Microplastic Effects on Thrombin–Fibrinogen Clotting Dynamics Measured via Turbidity and Thromboelastography

**DOI:** 10.3390/biom12121864

**Published:** 2022-12-13

**Authors:** Daniela Q. Tran, Nathan Stelflug, Abigail Hall, Tanmaye Nallan Chakravarthula, Nathan J. Alves

**Affiliations:** 1 Department of Emergency Medicine, Indiana University School of Medicine, Indiana University, Indianapolis, IN 46202, USA; 2 Weldon School of Biomedical Engineering, Purdue University, West Lafayette, IN 47907, USA

**Keywords:** microplastic, nanoplastic, thrombosis, thromboelastography, TEG, turbidity, fibrinogen, thrombin, polystyrene, coagulation

## Abstract

Micro/nanoplastics, whether manufactured or resulting from environmental degradation, can enter the body through ingestion, inhalation, or dermal pathways. Previous research has found that nanoplastics with diameters of ≤100 nm can translocate into the circulatory system in a dose-dependent manner and potentially impact thrombosis and hemostasis. To investigate the direct effects of microplastics on fibrin clot formation, a simplified ex vivo human thrombin/fibrinogen clot model was utilized. The 100 nm polystyrene particles (non-functionalized [nPS] and aminated [aPS]) were preincubated (0–200 µg/mL) with either thrombin or fibrinogen, and fibrin clot formation was characterized via turbidity and thromboelastography (TEG). When the particles were preincubated with fibrinogen, little effect was observed for aPS or nPS on turbidity or TEG up through 100 µg/mL. TEG results demonstrated a significant impact on clot formation rate and strength, in the case of nPS preincubated with thrombin exhibiting a significant dose-dependent inhibitory effect. In conclusion, the presence of microplastics can have inhibitory effects on fibrin clot formation that are dependent upon both particle surface charge and concentration. Negatively charged nPS exhibited the most significant impacts to clot strength, turbidity, and rate of fibrin formation when first incubated with thrombin, with its impact being greatly diminished when preincubated with fibrinogen in this simplified fibrin clot model.

## 1. Introduction

The widespread use of plastics across the globe seems unavoidable. Plastics provide companies and consumers several benefits such as improved costs, ease of manufacturing, and significant durability. The resulting increase in production and use of plastics has been a growing environmental concern for quite some time. One area of particular concern is the impact that the breakdown of plastics has on the environment and those that inhabit it. Due to the ubiquitous nature of plastic use in modern society and the exceptionally low, 8.6%, rate of recycling, it is estimated that there will be over 12,000 million metric tons of plastic waste in landfills and the environment by 2025 [1,2]. This trend has only been exacerbated by the increased utilization of single-use plastics that was driven by the COVID-19 pandemic in both consumer and medical markets [3,4,5]. A microplastic is generally considered to be any plastic whose diameter is less than 1 mm. Smaller plastic particles in the nanometer range can also be referred to as micro- or nano-plastics [6]. Particles that are released directly from the production of plastic particle additives for cosmetic products or plastic goods are known as primary microplastics [7,8]. Larger plastics can also break down once in the environment from mechanical and photo-degradation, and can form heterogenous mixtures of particles, known as secondary microplastics [9].

There are many types of plastic polymers utilized in industrial, medical, and consumer goods, such as polythene, polypropylene (PP), polyethylene terephthalate (PET), high-density polyethylene (HDPE), low-density polyethylene (LDPE), polyvinyl chloride (PVC), acrylic, and polystyrene (PS) [10]. It is common for plastics to possess a surface charge associated with the functionalization of plastic products during production to achieve desired material properties. Common surface modifications include amine, or positively charged surfaces (aPS), carboxyl, or negatively charged surfaces (cPS), and non-functionalized raw plastic surfaces that possess no intentional surface modification (nPS). Varied surface modifications can also result from the environmental degradation process itself and a single plastic particle can possess multiple different surface chemistries [11]. Polystyrene (PS), which is found in food packaging, CDs, toothbrushes, and office supplies, is both a common environmental pollutant and readily available commercially in a variety of particles sizes and surface chemistries. These qualities make PS a good laboratory model plastic for assessing microplastic impact on biological systems.

The presence of microplastics in the environment has grown considerably, such that widespread human exposure is seemingly inevitable. Microplastics have been found in air pollution, agricultural soil, table salt, drinking water, and other commercial products such as cosmetics and plastic packaged consumables [12,13,14,15]. The three primary pathways of microplastics exposure are inhalation, ingestion, and dermal exposure. In a study conducted by Cox et al., the level of human microplastic exposure is estimated to be between 74,000 and 114,000 particles per person, per year when including both ingestion and inhalation routes of exposure [16].

Plastic particles, 100 nm and under, have been shown to translocate from pulmonary and ingestion routes of exposures into tissues and the circulatory system in mammalian animal models [17,18,19,20,21]. Plastic has also recently been detected in human biological samples including stool, placenta, and blood [22,23,24]. This raises several questions about the impact of their presence on the various physiological processes that occur in the blood [25]. A primary concern regarding microplastics in the blood is how they interact with coagulation and fibrinolytic enzymes potentially impacting thrombosis and hemostasis.

Current research has focused on using modified PS particles in a range of bioavailable sizes in either direct exposure tests using animal models or in vitro coagulation tests such as platelet aggregation and prothrombin time [26,27,28,29]. Varying surface charge, dynamic and complex blood coagulation cascade, and experimentally diverse concentrations, sizes, and compositions of microplastics make deducing their impact on blood difficult. There are many aspects of the clotting cascade in which microplastics may play an activating or inhibiting role. Some studies involving isolated platelets and whole blood have been conducted previously that demonstrate inconsistent or inconclusive results [25].

**Figure 1:**
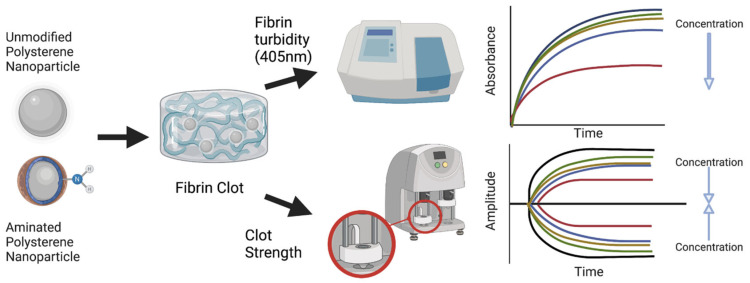
Determining microplastic surface charge and concentration effects on fibrin clot formation via turbidity (405 nm absorbance) and thromboelastography (TEG). Theoretical turbidity (absorbance) and TEG (amplitude) tracings are shown for reduced turbidity and reduced clot strength at increasing microplastic concentrations.

While previously utilized in vitro diagnostics have provided good insight into the clotting impacts of microplastics, they focus on clot formation dynamics and provide limited information regarding the structure or strength of the resulting clots themselves. Here, we investigate the dose-dependent effects of non-functionalized (nPS) and amine-functionalized (aPS) ~100 nm polystyrene particles in a simplified thrombin–fibrinogen clot model, tracking clot formation dynamics, structure, and strength via turbidity and thromboelastography (TEG) measurements [30,31] (Figure 1). The simplified thrombin–fibrinogen clotting model, utilizing purified human blood components, limits confounding variables that are introduced when using plasma or whole blood samples.

In an effort to study the impacts of microplastics on coagulation, we have elected to build from the ground up and investigate microplastic impacts on fibrinogen conversion by thrombin. Microplastic concentrations were assayed from 0 to 200 µg/mL to remain within a physiologically relevant microplastic range in blood. Tracking clot formation dynamics via both turbidity and TEG assays allows for insights to be gathered on the microplastic impact on clot structure, including fibrin fiber thickness and packing (turbidity), and ultimate clot strength (TEG) [32]. Additionally, both turbidity and TEG provide information regarding clotting dynamics that include time to clot initiation, rate of fibrin formation, and time to maximum clot formation. While this study does not independently recapitulate the entire coagulation system, it does provide significant insight into the effects of charge, surface coating, and which component that adsorbs to the particles first, influencing their ability to impact fibrin deposition.

## 2. Materials and Methods

### 2.1. Materials

The 100 nm aminated polystyrene particles (16586-5) and non-modified polystyrene (00876-15) particles were purchased from PolyScience (Warrington, PA, USA). Particles were stored at 4 °C and diluted 50-fold for use in turbidity and TEG assays. Plasminogen-depleted fibrinogen purified from human plasma (#341578) was purchased from Sigma-Aldrich (St. Louis, MO, USA) and stored lyophilized at −20 °C until rehydrated with PBS (7.4 pH, 0.01 M) directly prior to use. High activity thrombin from human plasma (#605195) was purchased from EMD Millipore (Burlington, MA, USA) and stored lyophilized. All fibrinogen and thrombin were thawed/hydrated immediately prior to testing and any material not used that day was discarded. Disposable TEG cups (#07-052) used for the TEG assays were purchased from Haemonetics (Boston, MA, USA).

### 2.2. Sample Preparation

On the day of the experiment, human fibrinogen was acclimated to room temperature and mixed with PBS (7.4 pH, 0.01 M) to a stock concentration of 12 mg/mL, 20 min prior to use. Concentration of fibrinogen was confirmed via absorbance at 280 nm (extinction coefficient of 513,400 L mol^−1^ cm^−1^, as provided by the manufacturer). Lyophilized human thrombin was stored in 20 U aliquots. On the day of the experiment, thrombin was acclimated to room temperature and then rehydrated with DI water to stock concentration of 20 U/mL. Solubilized thrombin was kept on ice prior to use.

### 2.3. Turbidity Assays

Turbidity assays were adapted from Zeng et al. [32]. Briefly, turbidity experiments were conducted on a Molecular Devices SpectraMax M5 plate reader using Softmax Pro 6.0 software (San Jose, CA, USA). A half area, 96-well plate (Corning # 3679) was used with final volume of 100 µL/well, run in quadruplicates. To test for thrombin and polystyrene interactions, thrombin was first incubated with either aPS or nPS for 1 min in 7.4 pH 0.01 M PBS. Clot initiation was accomplished by adding fibrinogen stock solution and mixed by pipetting to achieve a final concentration of 3 mg/mL fibrinogen and 1 U/mL thrombin in each well. To test for fibrinogen and polystyrene interactions, fibrinogen was first incubated with either aPS or nPS for 1 min in 7.4 pH 0.01 M PBS. Clot initiation was accomplished by adding thrombin stock solution and mixed by pipetting to achieve a final concentration of 3 mg/mL fibrinogen and 1 U/mL thrombin in each well. Turbidity was measured at 405 nm over 45 min in 10 s intervals at room temperature. Absorbance curves were evaluated for maximum turbidity (Turb^Max^), time to 90% maximum turbidity (Turb^Time^), and the slope of the linear phase.

### 2.4. Thromboelastography (TEG)

TEG assays were adapted from Zeng et al. [32]. Briefly, TEG assays were performed on a TEG^®^5000 Thromboelastograph Hemostasis Analyzer at 37 °C with TEG Analytical Software^®^ Version 4 (Haemonetics Corporation, Braintree, MA, USA) run in triplicate with one additional no-microplastic control cup per run. As noted, volumes of thrombin, fibrinogen, PBS, and microplastics were added to clear TEG cups (360 µL total). Sample preparation for TEG was carried out in the same manner as turbidity, keeping concentrations consistent across assays. TEG assays were allowed to run to completion. R-time, angle, maximum amplitude (TEG^max^), and time to maximum amplitude (TEG^time^) were recorded from the software.

### 2.5. Analysis

For Turb^max^, Turb^time^, slope, TEG^max^, TEG^time^, R-time, and angle, averages were taken from experimental quadruplicates (turbidity) or triplicates (TEG). Data are provided as mean ± standard deviations for the comparison of experimental results across assays for increasing microplastic concentrations, comparing surface charge and which clotting factor the particles were first exposed to.

## 3. Results and Discussion

### 3.1. Microplastic Characterization

Aminated (aPS) and non-functionalized (nPS) polystyrene particles were commercially sourced and validated prior to use. The nPS and aPS have reported mean sizes of 94 nm (+/−6.8 nm) and 110 nm (+/−4 nm) and were confirmed via dynamic light scattering to be 112.2 nm (PdI 0.039) and 107.6 nm (PdI 0.048), respectively (Supplemental Figure S1). It is important to note that the nPS particles do possess a negative charge due to the presence of sulfate esters, and this was confirmed on a Malvern Nano ZS90 with the nPS exhibiting a zeta potential of −55.2 ± 9.3 mV (Supplemental Figure S2). These ~100 nm particles were selected because they were available with both positive and negative charges and fell within the range of physiologically relevant sizes that can be found within the circulatory system. Experiments with nPS serve as a negative charge control to determine if effects on coagulation are related to surface charge (positive or negative), or potentially particle mass effect. Prior to use, the microplastic suspensions were inverted several times to ensure a homogenous microplastic mixture.

### 3.2. Turbidity

Turbidity measurements are a multiplexable optical plate assay used to assess how turbid a solution is, and can be applied to track fibrin clot formation over time. The resulting absorbance curves following the cleavage of soluble fibrinogen to insoluble fibrin by thrombin allow for characterization of the clotting rate via analysis of the slope of the initial linear phase. This linear fibrin conversation phase occurs leading to the gel point of the clot, before the sample reaches 15% of maximum turbidity [33]. Maximum clot turbidity (Turb^Max^) was determined and correlates to fibrin concentration as well as fibrin fiber thickness. Additionally, the time to reach 90% maximum turbidity (Turb^Time^) functions as an added rate measure extracted from the absorbance curves over time. In this way, turbidity can be utilized to determine if fibrin fiber packing is disrupted in the presence of microplastics, as well as to assess any kinetic microplastic effects, either slowing or accelerating the conversion of fibrinogen to fibrin.

Given that the microplastics were expected to impact the raw turbidity results, as they themselves are turbid at the concentrations tested, a calibration curve was performed using triplicates of either aPS or nPS alone, aPS or nPS plus thrombin, and aPS or nPS plus fibrinogen (Supplemental Figure S3). These absorbance values were then used to provide baseline corrections for all data seen throughout the turbidity results. The corresponding background values were chosen and analysis was carried out following baseline subtraction using the control absorbances of aPS or nPS.

#### 3.2.1. nPS Turbidity Effects on Fibrin Clotting

**Figure 2:**
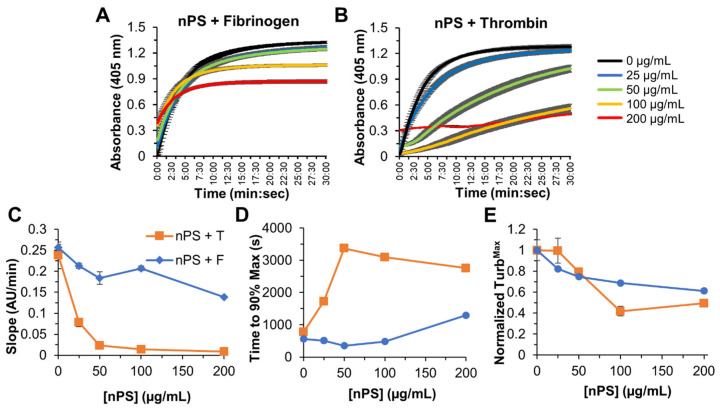
Dynamic turbidity curves over 30 min at concentrations of nPS microplastic from 0 to 200 µg/mL for preincubation of nPS + Fibrinogen ((**A**), nPS + F) and nPS + Thrombin ((**B**), nPS + T). (**C**) Change in absorbance per min over all concentrations. (**D**) Time to achieve 90% of the maximum absorbance. (**E**) Absolute maximum turbidity (Turb^Max^) at all concentrations, normalized and represented as fraction of the maximum turbidity of the no-microplastic control. Average and standard deviations of quadruplicates are plotted.

When increasing concentrations of nPS were preincubated with thrombin, there was up to a 27-fold decrease in initial velocity (slope), 2.4-fold decrease in Turb^Max^, and 4.4-fold increase in Turb^Time^ compared to the control, whereas preincubation of nPS with fibrinogen only resulted in a 1.9-fold decrease in initial velocity, 1.6-fold decrease in Turb^Max^, and 2.3-fold increase in Turb^Time^, at 200 µg/mL (Figure 2). These results with nPS are more dramatic than initially expected, as the literature indicates low reactivity of nPS in plate assays and animal exposure experiments exhibiting a pro-platelet aggregation effect [27,29,34]. While the particles are unmodified, they do possess a negative charge which likely contributes to the observed results, as surface charge is a known factor in how these particles effect coagulation [26,35,36]. In addition to the surprising nature of the nPS results, it is telling that the inhibitory effect was more pronounced when incubating with thrombin compared to preincubation with fibrinogen. In fact, though all concentrations reached a plateau in a 30 min time span when nPS was incubated with fibrinogen, only the lowest concentrations reached their max turbidity in this same time frame. This suggests that nPS is modulating the rate of thrombin converting fibrinogen to fibrin, likely by blocking or binding thrombin’s active site. This also suggests that once the microplastic particles are coated with protein, such as fibrinogen, their ability to bind thrombin and effect coagulation is greatly diminished.

#### 3.2.2. aPS Turbidity Effects on Fibrin Clotting

**Figure 3:**
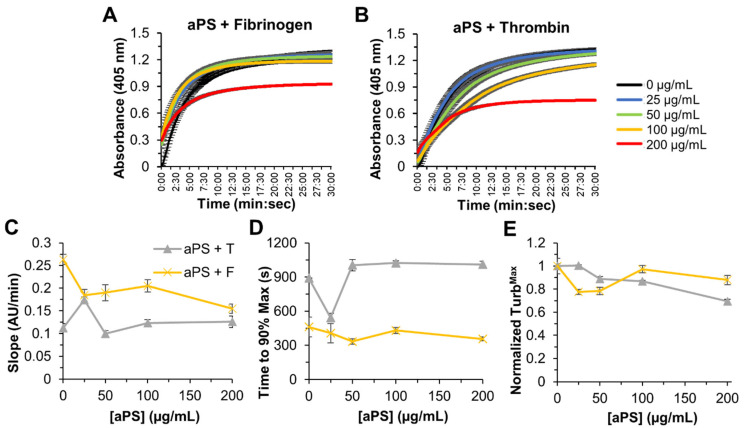
Dynamic turbidity curves over 30 min at concentrations of aPS microplastic from 0 to 200 µg/mL for preincubation of aPS + Fibrinogen ((**A**), aPS + F) and aPS + Thrombin ((**B**), aPS + T). (**C**) Change in absorbance per min over all concentrations. (**D**) Time to achieve 90% of the maximum absorbance. (**E**) Absolute maximum turbidity (Turb^Max^) at all concentrations, normalized and represented as fraction of the maximum turbidity of the no-microplastic control. Average and standard deviations of quadruplicates are plotted.

When increasing concentrations of aPS were preincubated with thrombin or fibrinogen, there was less than a 2-fold change in initial velocity, Turb^Max^, or Turb^Time^ (Figure 3). While there is little information available on aPS in turbidity measurements, in more complicated coagulation models, aPS has been shown to exhibit a stronger pro-coagulation effect then nPS. Combined with the results presented here, this suggests that the effect of aPS on coagulation is unlikely to occur at the thrombin conversation of fibrinogen to fibrin or at the fibrin-network formation level. Due to the complex nature of blood coagulation and fibrinolysis, it is likely that aPS in in vivo or plasma models interacts with the clotting cascade upstream of thrombin to impact downstream clot characteristics. The presence of aPS has been shown previously to increase platelet aggregation and thrombus generation in vivo. Taken together, these results indicate that aPS particles have little effect on the formation of the fibrin network directly [20,37].

### 3.3. Thromboelastography (TEG)

**Figure 4:**
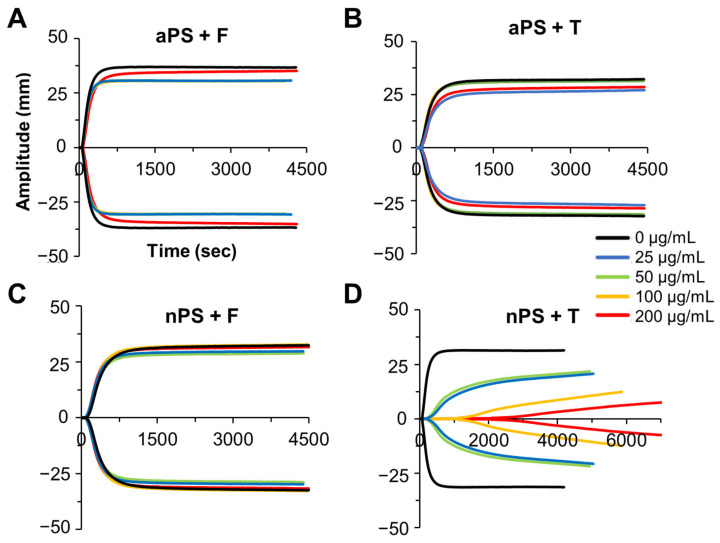
Dynamic TEG tracings over time aPS (**A**,**B**) and nPS (**C**,**D**). Representative singlet of TEG tracing for each condition are plotted. “+T” and “+F” indicate 1 min incubation with either thrombin or fibrinogen prior to clot initiation by addition of fibrinogen or thrombin, respectively.

TEG is a clinical instrument that takes a viscoelastic measurement of a clot throughout its dynamic formation. This compliments turbidity analysis by serving as a direct measure of clot strength, alongside inferred structural information from the optical qualities of a clot run under the same conditions. The TEG provides measures of R-time (time to clot formation), angle (speed of clot formation), and TEG^max^ (maximum clot strength achieved by the sample). As with turbidity, an additional measure of TEG^time^ (time to maximum clot strength) is also recorded for comparison. All nPS and aPS (0–200 µg/mL) premixtures with either fibrinogen or thrombin were assayed on Haemonetics TEG5000 instruments, tracking clot strength over time (Figure 4).

#### 3.3.1. nPS TEG Effects on Fibrin Clotting

**Figure 5:**
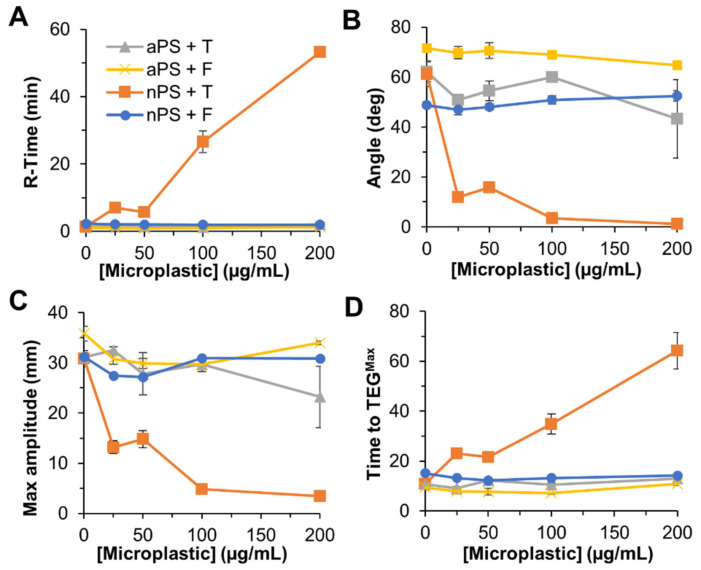
Summary of TEG results. (**A**) Clot initiation time (R-time). (**B**) Angle of tracing during linear stage of reaction, indicative of fibrin polymerization rate. (**C**) Maximum amplitude or maximum clot strength. (**D**) Time to reach maximum amplitude (min). Average and standard deviations of triplicates are plotted. “+T” and “+F” indicate 1 min incubation with either thrombin or fibrinogen prior to clot initiation by addition of fibrinogen or thrombin, respectively.

When increasing concentrations of nPS were incubated with fibrinogen, there was little change in TEG^max^, R-Time, angle, and TEG^time^ (Figure 4 and Figure 5). This was largely consistent with the observed turbidity results; however, more impact due to nPS was seen at the highest (200 μg/mL) particle concentration via turbidity than when assaying clot strength via TEG. When the nPS was incubated with thrombin first, there was a substantial dose-dependent effect on both clotting dynamics and ultimate clot strength, demonstrating a 9–fold decrease in TEG^max^, a 6-fold increase in TEG^time^, a 23-fold increase in R-time, and a 54-fold decrease in angle. While nPS preincubated with thrombin showed significant impact on clot turbidity (reducing maximum turbidity), the resulting microplastic impacts on clot strength were both larger in magnitude and occurred at lower concentrations of microplastic. All TEG values across all nPS TEG assays can be found in Supplemental Tables S1 and S2.

#### 3.3.2. aPS TEG Effects on Fibrin Clotting

When increasing concentrations of aPS were incubated with thrombin or fibrinogen, there was little impact observed in the resulting TEG^max^, TEG^time^, angle, and R-time (Figure 4 and Figure 5). This result is consistent with the turbidity data detailed in this manuscript. While this thrombin–fibrinogen clotting model displays limited effect from incubation with aPS, there are studies in the literature that show a pro-coagulation effect of aPS. Studies carried out by Nemmar et. al. observed increased human platelet aggregation as well as increased thrombus generation in hamsters when administering 50–100 µg/µL of 60 nm aPS [20]. While there are studies that suggest the presence of aPS induces a hypercoagulable state (increased thrombus formation), there is also another study that displayed an inhibitory effect on clotting with the application of aPS particles. Oslakovic et al. looked at increasing aPS concentrations in plasma and its effect on prothrombin time, a common clinical measure of coagulability [38,39]. In Oslakovic’s experiments, it was shown that higher concentrations of aPS increased prothrombin time, indicating impaired coagulation. The free clotting factors VII, IX, VIII, XI, protein S, and protein C were all found bound to isolated particles via Western blot, indicating significant surface adsorption of many coagulation factors. These models show conflicting results, similar to the thrombin–fibrinogen model discussed herein, but they are also examining different aspects of the coagulation pathway. The shift from pro-coagulation to anti-coagulation effects of aPS based on the protein sorption on the surface of the particles emphasizes the importance of these surface interactions and the assays utilized when determining coagulation effects. For a better understanding of plastic particle risk and mode of action, careful and systematic testing of different portions of the clotting cascade is needed. Taken together, the TEG and turbidity data utilizing aPS in this simplified thrombin–fibrinogen clotting model corroborates that the clots formed in the presence of aPS are not significantly affected, and it is likely that aPS impacts observed in prior studies are unrelated to the thrombin cleaving fibrinogen step within the clotting cascade. All TEG values across all aPS TEG assays can be found in Supplemental Tables S3 and S4.

### 3.4. Relationship between TEG and Turbidity

When considering the relationship between the turbidity and TEG results, thinner (or higher density fibrin fibers) are optically less turbid and contribute to a higher ultimate clot strength [33,40]. While nPS incubated with thrombin prior to clot initiation exhibited a decrease in turbidity, it also resulted in a significantly decreased clot strength. In this way, nPS appears to be inhibiting thrombin’s ability to convert fibrinogen to fibrin, which is contributing to both a reduced turbidity and reduced ultimate clot strength [41].

### 3.5. Use of Pristine Microplastics

As microplastic exposure is significantly linked to local environmental conditions and lifestyle, it is likely that people have widely different quantities of microplastic present systemically and that these levels may also rise and fall over time. A recent study quantifying plastic particles in human blood found that plastic was present in 17 out of 22 donors, with concentrations ranging from 1 to 12.3 µg/mL (1.6 ± 2.3 µg/mL on average) [24]. The study focused on only freely accessible particles of >700 nm in diameter flowing in the blood. Since particles <700 nm and particles adhered to vasculature or trapped within organs and hemostatic clots were not quantified, it is likely that these values are an underestimation of plastic in the blood. For this reason, the current study has focused on relatively high concentrations of pristine microplastics (25–200 µg/mL) to identify mechanistic effects of plastic on fibrinogen/thrombin interactions. There is currently little information regarding the dynamics of microplastics within the human body, and research in this area is critically needed. Additionally, in vitro data using human serum albumin (HSA) or patient plasma would help better tease out the effect that protein coated particles have on thrombosis. Particles are shown to rapidly gain a protein corona of HSA when exposed to plasma [42]. Additionally, the blood plasma concentration of serum albumin is 30–50 g/L [43], 10 times the concentration of fibrinogen, and prothrombin (circulating precursor of thrombin) is only found at 5–10 mg/dL [44]. It is unlikely that pristine microplastics, those that have no protein coat, will be present in the blood plasma due to the translocation requirement from inhalation through the lungs and ingestion through the gut prior to gaining access to the vasculature. At minimum, a realistic microplastic exposure will involve particles of varying size and composition with a protein corona, if not also a diverse array of surface chemistry modifications.

## 4. Conclusions

Leveraging this simplified fibrinogen/thrombin clotting model, we conclude that the presence of microplastics decreases ultimate turbidity and decreases the speed of fibrinogen conversion by thrombin, with non-modified negatively charged (nPS) particles having a greater effect than positively charged (aPS) particles. We also conclude that the presence of nPS decreases the strength of the formed clots, and that this inhibitory effect is due primarily to interactions between the particles and thrombin, and not interactions with fibrinogen. When first incubated with fibrinogen, followed by the addition of thrombin, there was little difference in ultimate clot strength, as determined by TEG, with either nPS or aPS. Protein coating of plastic particles modifies surface charge and can limit further microplastic particle interactions with other coagulation and fibrinolytic enzymes. One limitation of these findings is the use of only one size and shape of microplastic. The size of a particle determines surface area and curvature, which is shown to have an effect on coagulation factors upstream of thrombin [45]. While polystyrene is one of the most abundant microplastics found in the blood, the spherical, uniform nature of the particles used in this study does not provide the most accurate representation possible for secondary microplastics in the environment [46]. Still, insights from this current study will help future interpretation of experimental results in more complex clotting systems. There are advantages to simplifying the complexities of the clotting cascade to focus on individual clotting steps, as it allows these systems to be examined in isolation. However, additional factors are needed to fully understand how microplastic particles are affecting the structure and strength of clots under physiological conditions. Future experiments assaying various microplastics sizes and materials will be important for exploring surface area-based particle effects. Additionally, experiments should be carried out with more complex clotting models, strategically incorporating additional coagulation factors to deduce microplastic impact on clotting in a systematic manner, in order to assess what risk plastic particles present in the environment truly pose to human health.

## Data Availability

Data are contained within the article.

## Supplementary Materials

The following supporting information can be downloaded at: https://www.mdpi.com/article/10.3390/biom12121864/s1, Figures S1 and S2: Microplastic Particle Characterization; Figure S3: Microplastic Baseline Absorbance; Tables S1–S4: Summary of TEG Results for Each Microplastic-Fibrinogen-Thrombin Incubation Condition.
