# Supplementary material for: Microplastic Effects on Thrombin–Fibrinogen Clotting Dynamics Measured via Turbidity and Thromboelastography
Source: Biomolecules. 2022 Dec 13;12(12):1864. doi: 10.3390/biom12121864 (PMC9775992; doi:10.3390/biom12121864)
Supplement: Supplementary file 1 [file biomolecules-12-01864-s001.zip › biomolecules-2037018-supplementary.pdf]

Supplemental Material

Microplastic Effects on Thrombin-Fibrinogen Clotting Dynamics Measured via Turbidity and Thromboelastography

Daniela Q. Tran[1,†], Nathan Stelflug[2,†], Abigail Hall[1], Tanmaye Nallan Chakravarthula[1,3], Nathan J. Alves[1,3]*

1. Department of Emergency Medicine, Indiana University School of Medicine, Indiana University, Indianapolis, IN 46202, USA
2. Indiana University School of Medicine, Indiana University, Indianapolis, IN 46202, USA
3. Weldon School of Biomedical Engineering, Purdue University, West Lafayette, IN 47907, USA

[†]These authors contributed equally to this work

*Corresponding Author

[*] Corresponding author:
Nathan J. Alves, PhD
Indiana University School of Medicine
635 Barnhill Dr. Rm. 2063
Indianapolis, IN 46202, United States of America
E-mail address: nalves@iu.edu (N.J. Alves)

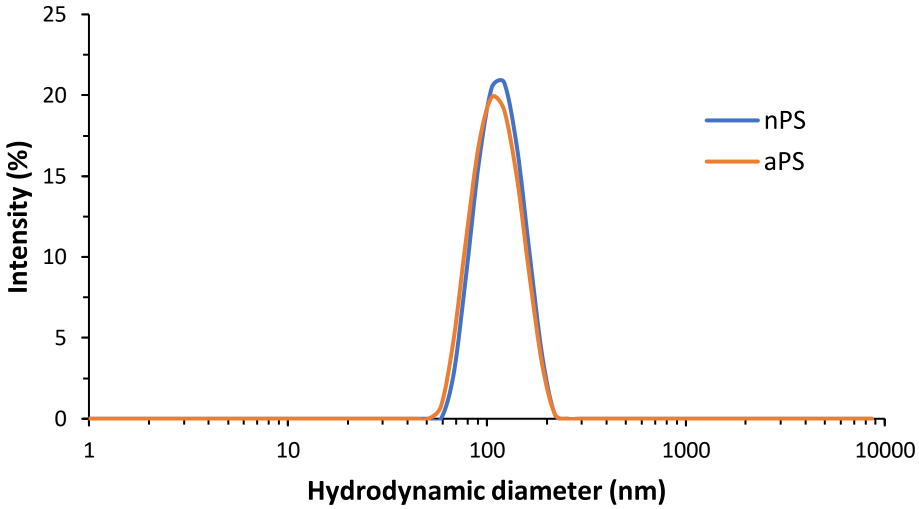

Supplemental Figure S1: The hydrodynamic diameter of nPS microplastics was 112.2 nm (PdI 0.039) and aPS microplastics was 107.6 nm (PdI 0.048). These were determined using a Zetasizer Nano ZS90 system (Malvern Instruments Ltd., Malvern, United Kingdom). Samples were diluted 100-fold in MilliQ deionized water, and the measurements were made at 25°C using 1 cm cuvettes. The results are the mean value from 3 runs.

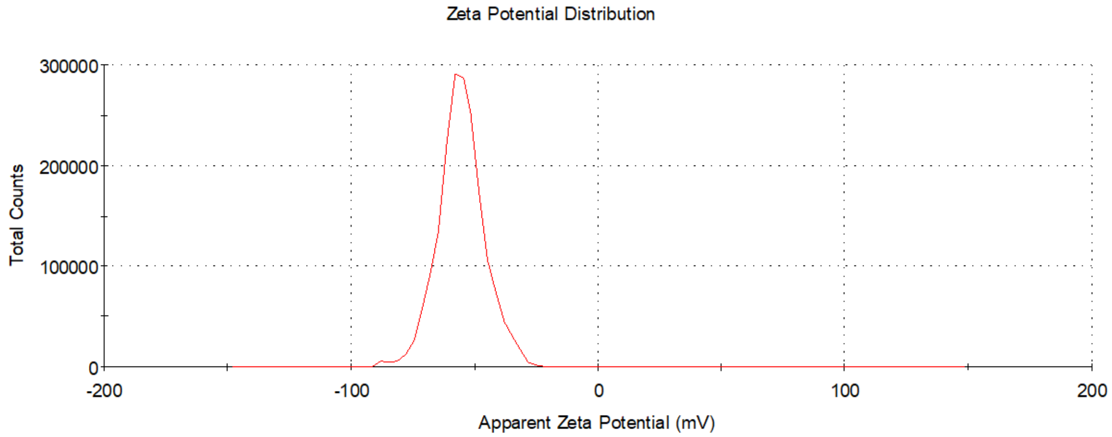

Supplemental Figure S2: The zeta potential of nPS microplastics was -55.2 ± 9.3 mV. This was determined using a Zetasizer Nano ZS90 system (Malvern Instruments Ltd., Malvern, United Kingdom). Samples were diluted 100-fold in MilliQ deionized water, and the measurements were made at 25°C. The results are the mean value from 3 measurements.

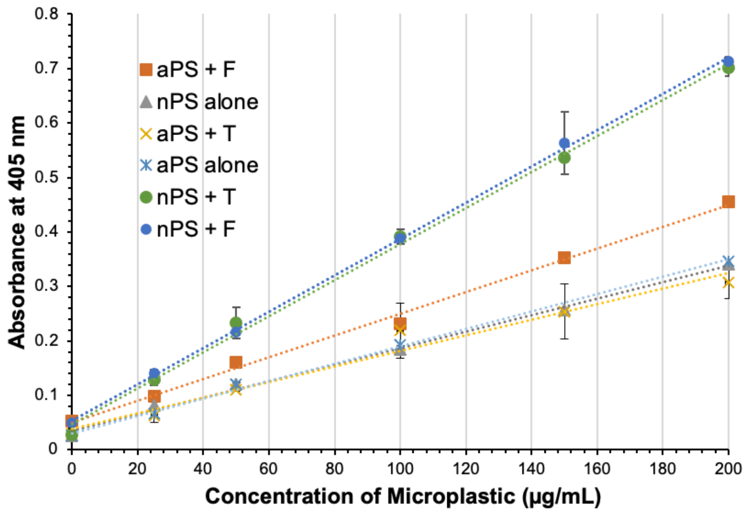

Supplemental Figure S3: The baseline absorbance of each microplastic or microplastic-reagent interaction at increasing concentrations of microplastics. Points represent the mean of triplicates ± standard deviation. The "+T" or "+F" indicates whether the microplastics were incubated with thrombin or fibrinogen, respectively.

Supplemental Table S1: Table of all TEG values **nPS+T**. Represented as averages ± standard deviation of triplicates or, in the case of the no microplastic controls, quadruplicates. Includes max amplitude, time to max amplitude, R time, and angle values used for Figure 5.

Concentration	0	25	50	100	200
SP(min)	1.15 ± 0.129	4.033 ± 0.208	3.5 ± 0.529	16.966 ± 1.625	0.966 ± 0.321
R(min)	1.4 ± 0.230	7.066 ± 0.642	5.733± 0.757	26.6 ± 3.157	53.233 ± 0.305
K(min)	2.8 ± 0.668				1.933 ± 0.305
Angle(deg)	61.225 ± 5.099	11.9 ± 1.417	15.8 ± 2.007	3.466 ± 0.568	1.133± 0.251
MA(mm)	30.875 ± 1.330	13.2 ± 1.3	14.8 ± 1.734	4.866 ± 0.115	3.466 ± 0.637
TMA(min)	10.375 ± 2.030	22.966 ± 0.115	21.566 ± 0.321	34.866 ± 4.061	64.2333 ± 1.069
G(d/sc)	2233.45 ± 139.770	760.4 ± 86.085	869.4 ± 118.528	257.133 ± 7.617	3008.36 ± 1045.51
E(d/sc)	44.675 ± 2.796	15.2 ± 1.734	17.4 ± 2.389	5.133 ± 0.152	60.2 ± 20.897
TPI(/sec)	8.375 ± 2.373	0 ± 0	0 ± 0	0 ± 0	16.466 ± 8.821
EPL(%)	0 ± 0	0 ± 0	0 ± 0	0 ± 0	0 ± 0
A30(mm)	30.875 ± 1.330	13.2 ± 1.3	14.8 ± 1.734	4.866 ± 0.115	36.9 ± 7.637
CL30(%)	100 ± 0	100 ± 0	100 ± 0	100 ± 0	100 ± 0
LY30(%)	0 ± 0	0 ± 0	0 ± 0	0 ± 0	0 ± 0
A60(mm)	30.875 ± 1.330	13.2 ± 1.3	14.8 ± 1.734	4.866 ± 0.115	36.9 ± 7.637
CL60(%)	100 ± 0	100 ± 0	100 ± 0	100 ± 0	100 ± 0
LY60(%)	0 ± 0	0 ± 0	0 ± 0	0 ± 0	0 ± 0
CLT(min)	-60.025 ± 0.736	-61.133 ± 0.057	-60.9 ± 0.529	-59.566 ± 2.223	-60.833± 1.484
A(mm)	31.65 ± 1.109	21.3 ± 1.777	21.166 ± 0.776	12.433 ± 0.850	37.7 ± 7.715
LTE(min)	227.5 ± 45	250 ± 0	250 ± 0	250 ± 0	302.166 ± 66.245

Supplemental Table S2: Table of all TEG values **nPS+F**. Represented as averages ± standard deviation of triplicates or, in the case of the no microplastic controls, quadruplicates. Includes max amplitude, time to max amplitude, R time, and angle values used for Figure 5.

Concentration	SP(min)	R(min)	K(min)	Angle(deg)	MA(mm)	TMA(min)	G(d/sc)	E(d/sc)	TPI(/sec)	EPL(%)	A30(mm)	CL30(%)	LY30(%)	A60(mm)	CL60(%)	LY60(%)	CLT(min)	A(mm)	LTE(min)
0	1.8 ± 0	2.3 ± 0.141	4.05 ± 0.173	48.7 ± 1.048	31.225 ± 1.087	15.175 ± 1.034	2269.75 ± 115.593	45.4 ± 2.348	5.65 ± 0.519	0 ± 0	31.225 ± 1.087	100 ± 0	0 ± 0	31.225 ± 1.087	100 ± 0	0 ± 0	-60.825 ± 2.209	33.175 ± 1.096	264 ± 16.166
25	1.533 ± 0.208	2.066 ± 0.152	4.533 ± 0.611	47 ± 2.116	27.466 ± 1.365	13.233 ± 1.059	1895.93 ± 128.550	37.9 ± 2.523	4.266 ± 0.808	0 ± 0	27.466 ± 1.365	100 ± 0	0 ± 0	27.466 ± 1.365	100 ± 0	0 ± 0	-61.333 ± 1.331	29 ± 1.385	250 ± 0
50	1.533 ± 0.251	2 ± 0.1	4.433 ± 0.351	48.066 ± 1.250	27.166 ± 1.069	12.2 ± 0.360	1867.166± 99.634	37.366 ± 1.985	4.266 ± 0.550	0 ± 0	27.166 ± 1.069	100 ± 0	0 ± 0	27.166 ± 1.069	100 ± 0	0 ± 0	-60.066 ± 1.814	28.533 ± 1.001	251.5 ± 2.022
100	1.533 ± 0.208	1.966 ± 0.115	3.766 ± 0.057	50.866 ± 1.692	30.933 ± 0.450	13.233 ± 0.503	2240.833 ± 46.911	44.8 ± 0.953	5.966 ± 0.251	0 ± 0	30.933 ± 0.450	100 ± 0	0 ± 0	30.933 ± 0.450	100 ± 0	0 ± 0	-60.333 ± 0.750	32.866 ± 0.208	250 ± 0
200	1.433 ± 0.251	1.933 ± 0.208	3.566 ± 0.321	52.433 ± 2.12	30.866 ± 2.010	14.166 ± 0.929	2236.7 ± 215.743	44.733 ± 4.285	6.333 ± 1.115	0 ± 0	30.866 ± 2.010	100 ± 0	0 ± 0	30.866 ± 2.010	100 ± 0	0 ± 0	-62.9 ± 0.964	32.6 ± 2.402	250 ± 0

Supplemental Table S3: Table of all TEG values **aPS+T**. Represented as averages ± standard deviation of triplicates or, in the case of the no microplastic controls, quadruplicates. Includes max amplitude, time to max amplitude, R time, and angle values used for Figure 5.

Concentration	0	25	50	100	200
SP(min)	1.033 ± 0.152	1.366 ± 0.115	1.2 ± 0.173	1.066 ± 0.450	1.5 ± 0.2
R(min)	1.3 ± 0.173	1.766 ± 0.057	1.633 ± 0.251	1.266 ± 0.416	2.066 ± 0.208
K(min)	2.5 ± 0.435	5.266 ± 0.115	3.666 ± 0.896	2.966 ± 0.152	4.1 ± 0
Angle(deg)	62.1 ± 4.026	50.733 ± 1.184	54.433 ± 4.006	59.966 ± 1.436	43.266 ± 15.672
MA(mm)	31.133 ± 0.901	32.766 ± 0.173	27.833 ± 4.188	29.766 ± 0.680	23.2 ± 6.148
TMA(min)	10.6 ± 2.029	13.333 ± 0.416	12.3 ± 0.7	10.566 ± 0.650	13 ± 1.252
G(d/sc)	2260.3 ± 91.580	1691.8 ± 13.982	1945.06 ± 387.755	2116.333 ± 69.689	1539 ± 502.757
E(d/sc)	45.233 ± 1.855	33.866 ± 0.288	38.9 ± 7.796	42.333 ± 1.361	30.8 ± 10.046
TPI(/sec)	9.1 ± 1.322	3.2 ± 5.44E-16	5.7 ± 2.170	7.133 ± 0.416	3 ± 2.598
EPL(%)	0 ± 0	0 ± 0	0.1 ± 0.173	0 ± 0	0 ± 0
A30(mm)	31.133 ± 0.901	25.3 ± 0.173	27.8 ± 4.246	29.766 ± 0.680	23.2 ± 6.148
CL30(%)	100 ± 0	100 ± 0	99.733± 0.461	100 ± 0	100 ± 0
LY30(%)	0 ± 0	0 ± 0	0.1 ± 0.173	0 ± 0	0 ± 0
A60(mm)	31.133 ± 0.901	25.3 ± 0.173	27.666 ± 4.476	29.766 ± 0.680	23.2 ± 6.148
CL60(%)	100 ± 0	100 ± 0	99.266 ± 1.270	100 ± 0	100 ± 0
LY60(%)	0 ± 0	0 ± 0	0.333 ± 0.577	0 ± 0	0 ± 0
CLT(min)	-61.8 ± 0.199	-60.8 ± 0.458	-62.4 ± 1.307	-59.933 ± 0.945	-63 ± 3.223
A(mm)	32 ± 1.410	27.266 ± 0.208	28.366 ± 5.000	31.333 ± 0.642	25.466 ± 5.692
LTE(min)	254.1 ± 7.101	250 ± 0	221 ± 50.229	250 ± 0	206.733 ± 74.940

Supplemental Table S4: Table of all TEG values **aPS+F**. Represented as averages ± standard deviation of triplicates or, in the case of the no microplastic controls, quadruplicates. Includes max amplitude, time to max amplitude, R time, and angle values used for Figure 5.

Concentration	SP(min)	R(min)	K(min)	Angle(deg)	MA(mm)	TMA(min)	G(d/sc)	E(d/sc)	TPI(/sec)	EPL(%)	A30(mm)	CL30(%)	LY30(%)	A60(mm)	CL60(%)	LY60(%)	CLT(min)	A(mm)	LTE(min)
0	0.825 ± 0.095	0.95 ± 0.129	1.625 ± 0.125	71.625 ± 0.921	35.825 ± 1.477	9.4 ± 0.697	2792.25 ± 177.537	55.825 ± 3.566	17.525 ± 2.145	0 ± 0	35.825 ± 1.477	100 ± 0	0 ± 0	35.8 ± 1.458	99.975 ± 0.049	0 ± 0	-61.025 ± 0.977	36.425 ± 1.241	260.825 ± 21.65
25	0.866 ± 0.115	1.033 ± 0.057	1.833 ± 0.152	69.7 ± 2.523	30.766 ± 1.123	7.833 ± 0.850	2223.06 ± 113.961	44.466 ± 2.302	12.433 ± 1.755	0 ± 0	30.766 ± 1.123	100 ± 0	0 ± 0	30.766 ± 1.123	100 ± 0	0 ± 0	-60.333 ± 1.404	31.066 ± 1.001	262.566 ± 12.126
50	0.666 ± 0.404	0.866 ± 0.404	1.666 ± 0.416	70.533 ± 3.181	31.633 ± 3.082	6.766 ± 1.950	2321.166 ± 338.647	46.433 ± 6.785	15.166 ± 7.008	1.066 ± 1.514	31 ± 2.325	98.166 ± 2.514	1.066 ± 1.514	30.7 ± 1.852	97.266 ± 3.900	1.766 ± 2.478	-60.766 ± 0.057	30.733 ± 1.850	250 ± 0
100	0.933 ± 0.152	1.033 ± 0.152	2 ± 0.173	68.933 ± 0.057	29.766± 1.497	7.133 ± 0.351	2121.133 ± 150.394	42.433 ± 3.023	10.666 ± 1.457	0 ± 0	29.766 ± 1.497	100 ± 0	0 ± 0	29.766 ± 1.497	100 ± 0	0 ± 0	-60 ± 1.609	30.233 ± 1.418	191.833 ± 50.227
200	1 ± 0.2	1.233 ± 0.057	2.133 ± 0.057	64.766 ± 1.446	34 ± 0.360	10.833 ± 0.513	2577.733 ± 42.354	51.566 ± 0.862	12.2 ± 0.458	0 ± 0	34 ± 0.360	100 ± 0	0 ± 0	34 ± 0.360	100 ± 0	0 ± 0	-61.366 ± 0.723	35.366 ± 0.378	226.633 ± 40.472
